# Extracts From *Hypericum hircinum* subsp. *majus* Exert Antifungal Activity Against a Panel of Sensitive and Drug-Resistant Clinical Strains.

**DOI:** 10.3389/fphar.2018.00382

**Published:** 2018-04-20

**Authors:** Noemi Tocci, Daniele Perenzoni, Duilio Iamonico, Francesca Fava, Tobias Weil, Fulvio Mattivi

**Affiliations:** ^1^Research and Innovation Centre, Fondazione Edmund Mach, San Michele all’Adige, Italy; ^2^Laboratory of Phytogeography and Applied Geobotany, Section Environment and Landscape, Department of Planning, Design, and Technology of Architecture, Sapienza University of Rome, Rome, Italy; ^3^Centre Agriculture Food Environment, University of Trento, Trento, Italy

**Keywords:** Hypericum, antifungal, Candida, cytotoxicity, LC-MS, phenolic compounds

## Abstract

During the last two decades incidences of fungal infections dramatically increased and the often accompanying failure of available antifungal therapies represents a substantial clinical problem. The urgent need for novel antimycotics called particular attention to the study of natural products. The genus *Hypericum* includes many species that are used in the traditional medicine to treat pathological states like inflammations and infections caused by fungi. However, despite the diffused use of *Hypericum*-based products the antifungal potential of the genus is still poorly investigated. In this study five *Hypericum* species autochthonous of Central and Eastern Europe were evaluated regarding their polyphenolic content, their toxicological safety and their antifungal potential against a broad panel of clinical fungal isolates. LC-MS analysis led to the identification and quantification of 52 compounds, revealing that *Hypericum* extracts are rich sources of flavonols, benzoates and cinnamates, and of flavan-3-ols. An in-depth screen of the biological activity of crude extracts clearly unveiled *H. hircinum* subsp. *majus* as a promising candidate species for the search of novel antifungals. *H. hircinum* is diffused in the Mediterranean basin from Spain to Turkey where it is traditionally used to prepare a herbal tea indicated for the treatment of respiratory tract disorders, several of which are caused by fungi. Noteworthy, the infusion of *H. hircinum* subsp. *majus* excreted broad antifungal activity against *Penicillium, Aspergillus* and non*-albicans Candida* isolates comprising strains both sensitive and resistant to fluconazole. Additionally, it showed no cytotoxicity on human cells and the chemical characterization of the *H. hircinum* subsp. *majus* infusion revealed high amounts of the metabolite hyperoside. These results scientifically support the traditional use of *H. hircinum* extracts for the treatment of respiratory tract infections and suggest the presence of exploitable antifungal principles for further investigations aimed at developing novel antifungal therapies.

## Introduction

The rising incidence of fungal infections is an emerging serious health problem, especially in cases of immune compromised patients caused either by diseases like AIDS, or by cancer therapies and prolonged antibiotic treatments (Lass-Flörl, 2009; Weil et al., 2017). Estimates of global mortality rates suggest that fungi are responsible for more deaths than either tuberculosis or malaria (Brown et al., 2012). Millions more suffer from severe illness caused by chronic or allergic fungal infections. *Candidiasis* is the most prevalent fungal disease worldwide difficult and extremely costly to treat (World Health Organization [WHO], 2014). The available antifungal drugs like polyenes, pyrimidines, azoles, and echinocandins often provoke significant toxicity including fever, nausea and vomiting, dose-limiting nephrotoxicity, liver toxicity, and controversial interactions with co-administered drugs (Dismukes, 2000; Zager, 2000). Another crucial problem is represented by the increasing rate of drug resistance that invalidates the clinical treatments. The alarming gap in the anti-fungal pipeline makes the discovery of new active compounds a priority for the scientific community. The strong request of new classes of compounds has renewed the interest in the screening of natural products (Abreu et al., 2012; Atanasov et al., 2016). Especially, ethnobotany represents an important guide in the search of novel antifungal agents. Due to their ability to synthesize a limitless variety of chemical structures, plants have represented a source of bioactive remedies for centuries (Arif et al., 2009). Despite of this, it is estimated that only a small proportion of plants are used by humans. Therefore, bioactivity screening programs and research on new plant-derived compounds are of prime importance for the search of novel leads. Plants belonging to the genus *Hypericum*, are characteristic components of the European flora. In particular, Europe and the Mediterranean basin represent a hot spot of diversity for *Hypericum* species (Nürk and Crockett, 2011). The best characterized species is *H. perforatum* L., which was already described by Hippocrates as a remedy against possession in the Ancient Greece (Dugoua et al., 2006). During the centuries it has been used for the treatment of various pathological states, like neurological disorders, wounds, inflammations, and infections. The use of *H. perforatum* extracts is so popular that this species is considered the most important medicinal plant of the 20th century (Gurib-Fakim, 2006). The German Commission E approved its usage for several indications, including treatment and post-therapy of acute and contused injuries, myalgia and first-degree burns as well as dyspeptic complaints (Arsic et al., 2010). In the Balkan Countries it is considered as one of the oldest remedies and in the Serbian folk medicine it is indicated for the treatment of cuts, burns, hemorrhoids and also as an antiseptic, for liver and stomach complaints, diarrhea, and gastric ulcers. Its sale represents a huge part of the overall herbal market (Crockett et al., 2011), exceeding $570 million/annum worldwide (Ayan et al., 2006). Regarding its antifungal potential, inhibitory properties against *Candida* spp., (Tocci et al., 2013) and fungal plant pathogens (Crockett et al., 2011) have been reported. Nevertheless, a growing number of scientific evidences underlines the importance to investigate other *Hypericum* species that might exert a more promising antifungal activity (Radulović et al., 2007; Stojanovic et al., 2013). In this regard the species *H. carinatum, H. myrianthum, H. linoides* (Barros et al., 2013), *H. garciae* (Tocci et al., 2018a) have been shown to have the potential to inhibit the growth of *Candida* spp. *Cryptococcus neoformans* and *Rhodotorula mucilaginosa* growth.

In Central and Eastern Europe several different *Hypericum* species co-occur and the area is considered a significant source of plant material for the market of herbal products (Raclariu et al., 2017).

Amongst others, *H. maculatum* Crantz subsp. *maculatum, H. hircinum* L. s. lat., *H. hirsutum* L., and *H. montanum* L., as plants used to treat wounds, skin inflammations and burns (Menkovic et al., 2011), as well as infections of the respiratory (Sagratini et al., 2008) and urinary tract (Sarić-Kundalić et al., 2010). These pathologies are often caused by fungi, with *Candida* as the main causative agent and by dermatophytes in the case of external infections (Borgers et al., 2005; Johnson, 2012; Ritchie and Eltahawy, 2014). To screen for new candidates suitable for the development of novel antimycotic solutions, we studied extracts deriving from the aerial parts of several *Hypericum* taxa (*H. perforatum* L., *H. maculatum* Crantz subsp. *maculatum, H. hircinum* L. s.lat., *H. hirsutum* L., and *H. montanum* L.) with respect to their chemical profile, their antifungal activity against a broad panel of clinical fungal isolates of the human gut and their toxicological safety on human skin fibroblasts and peripheral blood mononuclear cells (PBMCs).

## Materials and Methods

### Plant Material

Samples of *H. hirsutum* subsp. *hirsutum, H. hircinum* subsp. *majus, H. maculatum, H. montanum*, and *H. perforatum* have been collected during spring-summer of 2015 in the Alpine region (Northern-Eastern Italy). Species were identified according to the most recent Flora of Italy (Pignatti, 1982, 2017), nomenclature follows Bartolucci et al. (2018) and type specimens were deposited in the Department of Food Quality and Nutrition at the Fondazione Edmund Mach in San Michele all’Adige, Trento, Italy (Reference number: NT0001 *H. perforatum*, NT0002 *H. montanum*, NT0003 *H. maculatum*, NT0004 *H. hirsutum*, NT0005 *H. hircinum* subsp. *majus*).

### Preparation of Crude Plant Extracts

For each species, 500 mg of dried plant biomass, represented by stems and leaves, were powdered by grinding and extracted in the dark with methanol (drug/solvent ratio = 1:20 w/v) by maceration (3 × 24 h). *H. hircinum* subsp. *majus* was also subjected to extractions with boiling water (100°C) for 20 min, and ethanol 80% by maceration for 24 h. The obtained extracts were evaporated to dryness, weighed and stored at -20°C until analysis.

### Preparation of Samples for Chemical Analysis

Prior to analysis methanolic extracts were resuspended in 50% methanol/water and filtered using sterile (0.2 μm) PTFE filters.

### LC-MS Analysis

Analysis of phenolic metabolites was performed using a Waters Acquity UPLC system (Milford, MA, United States) which consists of a binary pump, an online vacuum degasser, an autosampler and a column compartment as previously described (Vrhovsek et al., 2012). Phenolic compounds were separated at a temperature of 40°C on a Waters Acquity HSS T3 column 1.8 μm, 150 mm × 2.1 mm (Milford, MA, United States). The mobile phase was composed of component A (0.1% formic acid in water) and component B (0.1% formic acid in acetonitrile). Acetonitrile was of LC-MS grade and was purchased from Sigma–Aldrich (St. Louis, MO, United States). Pure standards of hypericin from *Hypericum perforatum* ∼95% (HPLC), hyperoside and hyperforin were purchased from Sigma-Aldrich (St. Louis, MO, United States). Milli-Q water was used for the chromatography. The flow was set to 0.4 mL/min, and the gradient profile was: 0 min, 5% B; from 0 to 3 min, linear gradient to 20% B; from 3 to 4.3 min, isocratic 20% B; from 4.3 to 9 min, linear gradient to 45% B; from 9 to 11 min, linear gradient to 100% B; from 11 to 13 min, wash at 100% B; from 13.01 to 15 min, the re-equilibrated to the initial conditions of 5% B. Analysis of hypericin, hyperforin and hyperoside was performed using the same Waters Acquity UPLC system (Milford, MA, United States) and conditions, by means of a different gradient. The gradient profile in this case was: 0 min, 10% B; from 0 to 4,5 min, linear gradient to 100% B; from 4,5 to 12 min, isocratic 100% B; finally the column was re-equilibrated to the initial conditions of 10% B for 4 min. MRM transitions for hypericin, hyperforin and hyperoside are reported in Supplementary Table S1. Each sample was analyzed in triplicate. Samples were kept at 6°C during the analysis. Mass spectrometry detection for both methods was performed on a Waters Xevo TQMS (Milford, MA, United States) instrument equipped with an electrospray (ESI) source. Capillary voltage was 3.5 kV in positive mode and -2.5 kV in negative mode; the source was kept at 150°C; the desolvation temperature was 500°C; cone gas flow, 50 L/h; and desolvation gas flow, 800 L/h. Further MS parameters are reported in Vrhovsek et al., 2012.

### Data Analysis

Quantification was done using Waters MassLynx 4.1 and TargetLynx software.

### Microorganisms and Media

For antifungal susceptibility testing, the reference strain *C. albicans* ATCC MYA-2876 from the American Type Culture Collection (ATCC), Rockville, MD, United States was used. The human gut clinical isolates, *C. albicans* YN7, *C. parapsilosis* YB1, *C. parapsilosis* YB3 (Di Paola et al., unpublished), *C. albicans* YHS254, *C. albicans* YHS89, *C. lusitaniae* YHS217, *C. lusitaniae* YHS72, *C. parapsilosis* YHS133, *C. parapsilosis* YHS312, *C. parapsilosis* YHS301, *Aspergillus glaucus* YHS165, *Penicillium paneum* YHS245 (Strati et al., 2016), *C. glabrata* MFB004, *C. tropicalis* MFB035-1, *C. tropicalis* RTT037-3 (Strati et al., unpublished) were tested for their susceptibility against the applied drugs. All strains were grown on Sabouraud agarized medium for 48 h at 30°C before testing.

### Antifungal Susceptibility Testing

*Candida* strains were tested for their susceptibility to *Hypericum* spp. extracts (7 dilution series, ranging from 500 to 8 μg/ml) following the European Committee for Antimicrobial Susceptibility Testing protocol (EUCAST DEFINITIVE DOCUMENT EDef 7.2 Revision; European Committee for Antimicrobial Susceptibility Testing, 2012). Briefly, cells were grown in RPMI1640 medium supplied with 2.0% glucose, counted and inoculated at a concentration of 1–5 × 10^5^ CFU/ml. MIC_50_ and MIC_90_ values were determined at 530 nm using a spectrophotometer, as the lowest concentration of the drug that resulted in ≥50% and ≥90% inhibition of growth relative to the growth control after 48 h incubation.

### PBMC Isolation

Peripheral blood mononuclear cells were obtained from buffy coat of healthy donors (as approved by the Ethical Committee of the local health centre, Azienda Provinciale per I Servizi Sanitari, Provincia Autonoma di Trento) within 4 h from sample collection. PBMC isolation was performed using Lympholite (CEDARLANE) density gradient (1.077 g/liter, pH 6,9), according to manufacturer’s instructions, and incubated in complete nutrient RPMI 1640 medium (Euroclone) supplemented with glutamine (2 mol/L), 10% fetal calf serum (FCS) and antibiotics (10000 U/ml Penicillin, 10000 μg/ml Streptomycin) (Biological Industries), at 37°C and 5% CO2.

### Cytotoxicity Assay

The cytotoxicity of *H. hircinum* subsp. *majus* infusion, methanolic and hydro-alcoholic extracts was tested using the WST-8 (4-[3-(2-methoxy-4-nitrophenyl)-2-(4-nitrophenyl)-2*H*-5-tetrazolio]-1,3-benzene disulfonate sodium salt) conversion assay. The cytotoxicity of the extracts was evaluated on two normal cell lines, namely PBMC and human foreskin fibroblast Hs27. Cells were seeded in 96-well culture plates at concentration of 5000 cells/well and incubated for 24 h before treatment with *H. hircinum* subsp. *majus* extracts. Plant extracts were diluted in DMSO and added to a final concentration of 500 μg/ml (final volume of DMSO 0,5% v/v). To measure the cytotoxic effects 10 μl of Cell Counting Kit-8 (Sigma-Aldrich) (containing the WST-8 solution) were added to each well and incubated for 2–4 h for fibroblasts and PBMCs, respectively, at 37°C before reading the absorbance at 450 nm.

The results were analyzed by Student’s *t*-test. The data are expressed as the percentage of viability and standard error of the mean.

### Microscopy

To estimate the impact of plant extracts on cell morphology and health, cultures were visualized and recorded using an inverted microscope Motic AE31 equipped with a camera Moticam 580 5.0 MP.

## Results

### Extract Composition

The extracts have been chemically characterized by the identification and quantification of 52 low molecular weight polyphenols belonging to the classes: flavonols, benzoates and cinnamates, chalcones, flavan-3-ols, flavones, stilbens, coumarins, phloroglucinols, and naphtodianthrones (**Table 1**). LC-MS analysis revealed that species were characterized by different content of the detected metabolites, yet in all of the extracts, quercetin derivatives represented the most prevalent group of compounds.

**Table 1:** Analysis of phenolic compounds in the crude extracts of *Hypericum* species.

	*H. hirc majus*	*H. hirs*	*H. mac*	*H. mont*	*H. perf*
	
	μg/g extract				
Benzoates and cinnamates	427,04 ± 47,1	45,9 ± 3,65	243,22 ± 1,14	390,72 ± 6,17	112,6 ± 10,5
2,6-di-OH-benzoic acid	nd^∗^	nd^∗^	nd^∗^	nd^∗^	nd^∗^
3,5-dihydroxybenzoic acid	1,78 ± 0,013	1,98 ± 0,012	1,47 ± 0,013	0,98 ± 0,009	0,96 ± 0,016
Caffeic acid	nd^∗^	nd^∗^	nd^∗^	nd^∗^	nd^∗^
Cinnamic acid	nd^∗^	nd^∗^	nd^∗^	nd^∗^	nd^∗^
Ellagic acid	5,83 ± 0,073	3,29 ± 0,088	7,22 ± 0,114	6,25 ± 0,10	2,18 ± 0,054
Ferulic acid	0,06 ± 0,003	0,38 ± 0,003	0,54 ± 0,012	nd^∗^	0,26 ± 0,006
Gallic acid	0,70 ± 0,028	1,07 ± 0,008	0,51 ± 0,013	0,73 ± 0,032	0,42 ± 0,0013
*p*-coumaric acid	0,08 ± 0,001	0,6 ± 0,019	0,26 ± 0,009	0,25 ± 0,002	0,23 ± 0,007
*p*-OH-benzoic acid	nd^∗^	nd^∗^	nd^∗^	nd^∗^	nd^∗^
Vanillic acid	nd^∗^	nd^∗^	nd^∗^	nd^∗^	nd^∗^
Chlorogenic acid	323,03 ± 4,946	8,51 ± 0,450	nd^∗^	nd^∗^	18,92 ± 0,949
Cryptochlorogenic acid	0,58 ± 0,009	0,12 ± 0,007	1,32 ± 0,048	4,44 ± 0,025	0,72 ± 0,01
Neochlorogenic acid	95,07 ± 0,194	29,95 ± 0,032	231,90 ± 0,112	378,07 ± 0,449	88,91 ± 0,204
Flavonols	9393,64 ± 50,3	7470,05 ± 28,01	6555,52 ± 30,21	5284,31 ± 13,25	7189,19 ± 47,26
Hyperoside	9306 ± 385	7430 ± 211	6240 ± 488	4940 ± 66,3	6880 ± 469
Quercetin	2,28 ± 0,043	9,67 ± 0,063	45,44 ± 0,159	14,35 ± 0,085	5,13 ± 0,011
Quercetin-3-arabinoside	nd^∗^	0,19 ± 0,005	32,40 ± 0,099	23,19 ± 0,047	132,86 ± 1,522
Quercetin-3-glucoside	43,56 ± 0,536	18,12 ± 0,529	226,89 ± 1,948	235,33 ± 1,712	14,28 ± 0,479
Quercetin-3-glucuronide	28,77 ± 0,212	nd^∗^	nd^∗^	nd^∗^	2,54 ± 0,02
Quercetin-3-sulfate	11,91 ± 0,026	2,22 ± 0,018	4,64 ± 0,024	0,69 ± 0,004	34,82 ± 0,024
Rutin	nd^∗^	2,47 ± 0,031	3,19 ± 0,015	0,86 ± 0,006	118,89 ± 0,197
Kaempferol	0,025 ± 0,001	1,73 ± 0,007	0,43 ± 0,005	0,11 ± 0,001	0,19 ± 0,001
Kaempferol-3-glucuronide	0,48 ± 0,0151	nd^∗^	nd^∗^	nd^∗^	0,05 ± 0,001
Kaempferol -3-glucoside	0,38 ± 0,012	2,38 ± 0,071	2,16 ± 0,038	1,18 ± 0,031	0,33 ± 0,009
Syringetin-3-glucoside	nd^∗^	nd^∗^	nd^∗^	nd^∗^	nd^∗^
Iso-rhamnetin	nd^∗^	nd^∗^	0,13 ± 0,003	nd^∗^	nd^∗^
Iso-rhamnetine-3-glucoside	nd^∗^	0,08 ± 0,004	nd^∗^	nd^∗^	nd^∗^
Myricetin	0,23 ± 0,004	3,19 ± 0,014	0,24 ± 0,003	0,29 ± 0,004	0,10 ± 0,002
Flavones	0,65 ± 0,036	2,215 ± 0,016	1,175 ± 0,006	3,035 ± 0,014	0,45 ± 0,010
Luteolin	trace	1,93 ± 0,011	0,79 ± 0,004	2,52 ± 0,009	0,22 ± 0,006
Luteolin 7-O-glucoside	0,65 ± 0,036	0,28 ± 0,005	0,38 ± 0,002	0,51 ± 0,005	0,18 ± 0,004
Flavanones	0,44 ± 0,009	1,43 ± 0,031	0,58 ± 0,018	2,3 ± 0,009	0,82 ± 0,016
sNaringenin	0,05 ± 0,004	0,18 ± 0,006	nd^∗^	0,07 ± 0,002	0,10 ± 0,004
Naringenin-7-glucoside	0,39 ± 0,005	1,25 ± 0,025	0,58 ± 0,018	2,23 ± 0,007	0,72 ± 0,012
Chalcones	0,11 ± 0,006	0,42 ± 0,005	0,32 ± 0,008	nd^∗^	0,31 ± 0,005
Phlorizin	0,11 ± 0,006	0,42 ± 0,005	0,32 ± 0,008	nd^∗^	0,31 ± 0,005
Coumarins	0,23 ± 0,0032	0,07 ± 0,002	0,14 ± 0,004	0,55 ± 0,005	0,22 ± 0,002
Esculin	0,23 ± 0,0032	0,07 ± 0,002	0,14 ± 0,004	0,55 ± 0,005	0,22 ± 0,002
Flavan-3-ols	53,12 ± 1,24	74,12 ± 3,12	100,09 ± 4,81	58,28 ± 1,53	59,47 ± 2,47
Catechin	21,94 ± 0,609	5,25 ± 0,034	3,68 ± 0,114	1,79 ± 0,048	4,21 ± 0,161
Epicatechin	19,28 ± 0,212	40,21 ± 0,258	68,52 ± 0,029	53,61 ± 0,166	43,76 ± 0,17
Epigallocatechin	3,31 ± 0,030	0,42 ± 0,014	0,52 ± 0,008	1,44 ± 0,031	0,82 ± 0,025
Gallocatechin	2,21 ± 0,032	nd^∗^	nd^∗^	nd^∗^	nd^∗^
Procyanidin B_1_	1,37 ± 0,007	0,94 ± 0,025	0,89 ± 0,018	nd^∗^	0,42 ± 0,005
Procyanidin B_2_	5,01 ± 0,091	27,3 ± 0,521	26,48 ± 0,770	1,44 ± 0,287	10,26 ± 0,111
Stilbenoids	0,06 ± 0,001	0,91 ± 0,015	2,24 ± 0,009	0,99 ± 0,008	3,32 ± 0,052
*cis*-piceid	0,06 ± 0,001	0,91 ± 0,015	2,24 ± 0,009	0,99 ± 0,008	3,32 ± 0,052
Phloroglucinols	nd^∗^	nd^∗^	nd^∗^	20 ± 1,91	8050 ± 565
Hyperforin	nd^∗^	nd^∗^	nd^∗^	20 ± 1,91	8050 ± 565
Naphtodianthrones	nd^∗^	30 ± 1,79	20 ± 1,71	40 ± 2,67	150 ± 25,1
Hypericin	nd^∗^	30 ± 1,79	20 ± 1,71	40 ± 2,67	150 ± 25,1

*H. hirs majus, H. hircinum subsp. majus; H. hirs, H. hirsutum; H. mac, H. maculatum; H. mon, H. montanum; H. perf, H. perforatum. ^∗^nd, not detected.*

Quercetin and quercetin glycosides ranged from 5,21 mg/g in *H. montanum* to 9,4 mg/g in *H. hircinum* subsp. *majus*, with hyperoside being the major compounds in the extracts representing the 45,48% of the detected compounds in *H. perforatum* and more than the 99% in the other *Hypericum* species. Quercetin-3-glucoside was abundant in *H. maculatum* and in *H. montanum*, while quercetin 3-rhamnoside was highly present in *H. perforatum* (132,9 μg/g). Quercetin-3-glucuronide only occurred in *H. hircinum* subsp. *majus* (28,8 μg/g) and in *H. perforatum* (10-fold less concentrated). Chlorogenic acid derivatives (chlorogenic acid, neochlorogenic acid, and cryptochlorogenic acid) ranged from 38 μg/g for *H. hirsutum* and *H. perforatum* to 456,7 μg/g in *H. hircinum* subsp. *majus*. Also the flavan-3-ols were relatively abundant ranging from 53,12 μg/g in *H. hircinum* subsp. *majus* to 100,12 μg/g in *H. maculatum*, with epicatechin being the principal compound of this class (from 19 μg/g in *H. hircinum* subsp. *majus* up to 68,5 μg/g in *H. maculatum*).

### Antifungal Activity of *Hypericum* Crude Extracts

*Hypericum* extracts were screened against a panel of clinical *Candida* isolates (**Table 2** and Supplementary Table S2) and the results are hereafter given as GM of MIC values. The screening unveiled that *H. hircinum* subsp. *majus* crude methanolic extract had a strong anti-*Candida* activity against all tested fungal strains, with inhibition properties against *C. parapsilosis* (MIC_50_ 53,5 μg/ml) in the same magnitude of fluconazole (MIC_50_ 22,63 μg/ml).

**Table 2:** Antifungal activity of *Hypericum* species.

Species (strain no.)	*H. hircinum*		*H. maculatum*		*H. montanum*		*H. perforatum*		*H. hirsutum*		Fluconazole	
	MIC_50_	Range	MIC_50_	Range	MIC_50_	Range	MIC_50_	Range	MIC_50_	Range	MIC_50_	Range
*C. albicans* (2)	63,25	32–125	250	250	500	500	353,3	250–500	500	500	0,35	0,125–1
C. *parapsilosis* (2)	32	32	500	500	>500	>500	>125	125–500	>500	500	48	32–64
*C. tropicalis* (2)	353,55	>500	>500	>500	>500	>500	>500	>500	>500	>500	>64	>64
*C. lusitaniae* (1)	16	125	64	64	125	125	125	125	64	64	0,5	0,5
*C. glabrata* (1)	250	>500	>500	>500	>500	>500	>500	>500	>500	>500	0,13	0,13

*Data are expressed as GM in μg/ml.*

All extracts exerted antifungal properties against *C. albicans* and *C. lusitaniae* while only *H. hircinum* subsp. *majus* was also active against *C. tropicalis* and *C. glabrata* (MIC_50_ 353,55 and 16 μg/ml, respectively).

Also, with respect to the to the MIC_90_ values, *H. hircinum* subsp. *majus* extract showed higher inhibitory properties than the other tested extracts (Supplementary Table S2).

### Antifungal Activity of *H. hircinum* subsp. *majus* Extracts

The antifungal activity of *H. hircinum* subsp. *majus* was further investigated against a broader panel of *Candida*, including fluconazole resistant strains, and other fungi (**Table 3** and Supplementary Table S3). In addition to crude methanolic extract, infusion and hydroalcoholic extracts have been tested. Notably, all *H. hircinum* subsp. *majus* extracts showed antifungal properties and, in particular a remarkable activity against fluconazole resistant *C. parapsilosis* and *C. tropicalis* strains. The methanolic extract showed the most pronounced cytotoxic properties. Yet, in general the antifungal activity of the infusion outcompeted those of the hydroalcoholic extract, also with respect to the MIC_90_ values.

**Table 3:** Antifungal activity of *H. hircinum* subsp. *majus*.

Species (strain no.)	Methanol		Ethanol 80%		Infusion		Fluconazole	
	MIC_50_	MIC_90_	MIC_50_	MIC_90_	MIC_50_	MIC_90_	MIC_50_	MIC_90_
*C. albicans* (4)	31,81	≥149,5	75,21	>420,4	63,25	>250	4	≥9,5
*C. tropicalis* (2)	353,55	>500	353,55	>500	177	>500	>64	>64
*C. parapsilosis* (5)	53,5	≥250	250	≥500	95,18	≥300	≥60	>64
*C. lusitaniae* (2)	16	89,4	63,25	250	32	177	0,35	1,5
*C. glabrata* (1)	16	64	64	250	64	250	0,13	1
*Aspergillus glaucus* (1)	>500	>500	>500	>500	250	>500	0,25	16
*Penicillium paneum* (1)	>500	>500	>500	>500	64	500	0,13	4

*Data are expressed as GM in μg/ml.*

### Cytotoxicity of *H. hircinum* subsp. *majus* Extracts

**FIGURE 1:**
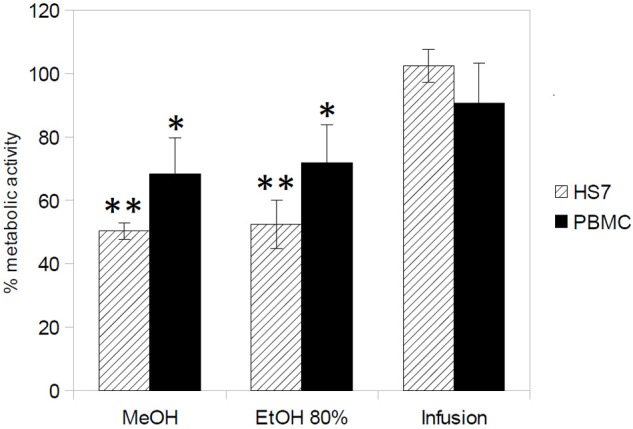
Cytotoxicity of *H. hircinum* subsp. *majus* extracts (measured using the WST-8 conversion assay). The metabolic activity is given as the endpoint of toxicity in human peripheral mononucleate cells (PBMC) and human skin fibroblast cell line (Hs27), following 24 h exposure to *H. hircinum* subsp. *majus* extracts. Values represent standard error of the mean (*n* = 3); Asterisks denote statistically significant differences from control (^∗^*p* < 0.05 and ^∗∗^*p* < 0.01).

The cytototoxicity of *H. hircinum* subsp. *majus* extracts was tested on PBMC and human skin fibroblast Hs27 cell lines by using the WST-8 conversion assay. The extracts were tested at a dose of 500 μg/ml, which corresponds to a 1- (for *C. tropicalis*) to 10-fold higher concentration than those showing inhibition of 50% of *Candida* growth. As shown in **Figure 1**, only the plant infusion showed no cytotoxic effect on both human cell lines, while treatment with methanolic and hydroalcoholic extracts inhibited cell viability by 50% for Hs27 cell line and by 65–70% for PBMCs.

**FIGURE 2:**
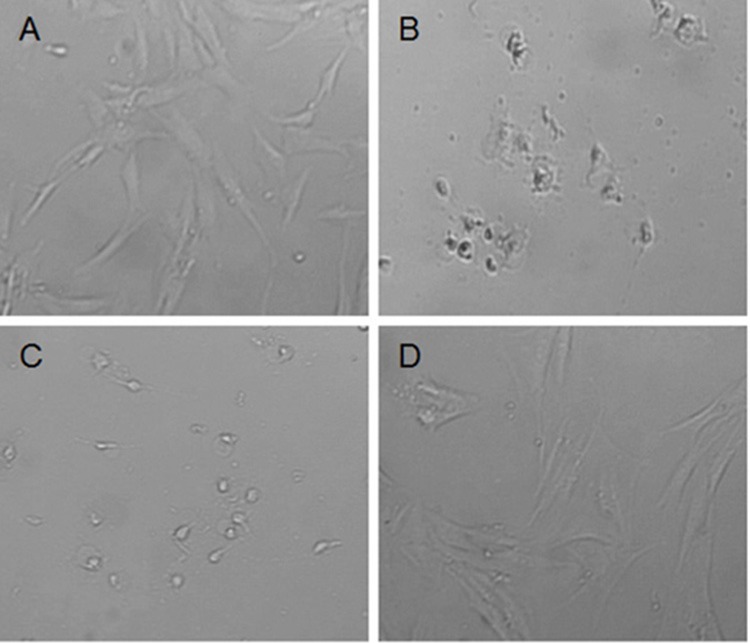
Morphology change of normal fibroblast Hs27 after 24 h treatment with *H. hircinum* subsp. *majus* extracts (observed under inverted microscope, magnification 100×): **(A)** control (DMSO), **(B)** methanolic extract, **(C)** hydroalcoholic extract, and **(D)** infusion.

The effect of *H. hircinum* subsp. *majus* on human cells was also observed microscopically (**Figure 2**). The imagines confirmed the cytotoxicity data. Fibroblast treated with plant infusion showed a normal phenotype, while under treatment with methanolic and hydro-alcoholic extracts the cell morphology was markedly altered. The same alteration was observed for PBMCs (data not shown).

### Chemical Composition of *H. hircinum* susbps. *majus* Infusion

The chemical composition of the aqueous extract of *H. hircinum* subsp. *majus* is reported in **Table 4**. The analysis revealed that the infusion process allowed the extraction of higher amounts of phenols in comparison to the extraction by maceration in methanol. The aqueous extract was mainly characterized by derivatives of quercetin (24,18 mg/g), of chlorogenic acid (4,51 mg/g) and by flavan-3-ols (2,26 mg/g). Hyperoside was the most abundant compound, representing the 2,23% of the extract. Abundant were also quercetin-3-glucoside (1,65 mg/g), neochlorogenic acid (2,61 mg/g), chlorogenic acid (1,68 mg/g), procyanidin B2 (0,89 mg/g), epicatechin (0,59 mg/g) and procyanidin B1(0,40 mg/g).

**Table 4:** Analysis of phenolic compounds in the infusion of *H. hircinum* subsp. *majus.*

Compound	Amount (μg/g extract)	Compound	Amount (μg/g extract)
2,6-di-OH-benzoic acid	nd^∗^**^∗^**	Kaempferol	1,08 ± 0,15
3,5-dihydroxybenzoic acid	7,08 ± 0,79	Kaempferol -3-glucoside	7,89 ± 1,09
Caffeic acid	44,62 ± 4,97	Kaempferol-3-glucuronide	104,41 ± 14,49
Coniferyl alcohol	nd^∗^	Iso-rhamnetin	0,78 ± 0,11
Gallic acid	15,74 ± 2,18	Iso-rhamnetine-3-glucoside	nd^∗^
Cinnamic acid	6,10 ± 0,85	Syringetin-3-glucoside	3,39 ± 0,46
*p*-coumaric acid	nd^∗^	Myricetin	7,68 ± 1,07
*p*-OH-benzoic acid	8,97 ± 1,24	Luteolin	3,51 ± 0,22
Vanillic acid	1,29 ± 0,18	Luteolin 7-O-glucoside	1,77 ± 0,24
Cryptochlorogenic acid	229,7 ± 31,88	Naringenin	nd^∗^
Chlorogenic acid	1683 ± 233,7	Esculin	21,28 ± 2,95
Neochlorogenic acid	2615 ± 362,9	Phlorizin	6,57 ± 0,91
Hyperoside	22203 ± 380	Catechin	282,2 ± 2,71
Quercetin	164,38 ± 15,52	Epicatechin	586,51 ± 68,05
Quercetin-3-arabinoside	nd^∗^	Epigallocatechin	55,77 ± 7,74
Quercetin-3-glucoside	1647 ± 65,03	Gallocatechin	48,92 ± 9,72
Quercetin-3-glucuronide	104,41 ± 14,92	Procyanidin B_1_	401,6 ± 55,74
Quercetin-3-sulfate	nd^∗^	Procyanidin B_2_	886,16 ± 108,77
Rutin	nd^∗^	*cis*-piceid	1,37 ± 0,19

*Data are means of three independent experiments, ^∗^nd, not detected.*

## Discussion

Plant-based treatments of the traditional medicine could bring forth new solutions for antifungal therapies. In particular polyphenol-rich plants represent an import source of bioactive products for human health (Marchelak et al., 2017) including antifungal treatments. Among polyphenols, flavan-3-ols, flavonols and tannins show a high antimicrobial potential (Daglia, 2012). Flavan-3-ols isolated from green and black tea exert important anti-*Candida* activity (Hirasawa and Takada, 2004; Sitheeque et al., 2009), ellagitannins obtained from *Punica granatum* inhibit the growth of plant pathogenic fungi (Glazer et al., 2012), the flavonols myricitrin and fisetin are considered effective agents against *Candida glabrata* and *Cryptococcus neoformans*, respectively (Salazar-Aranda et al., 2015; Reis et al., 2016). The mechanisms of action of phenols against *Candida* have been intensively studied and include inactivation of enzyme production (Evensen and Braun, 2009) and anti-biofilm effects (Evensen and Braun, 2009; Shahzad et al., 2014). Species belonging to the *Hypericum* genus are able to synthesize a broad array of such bioactive phenolic compounds, mainly as products of the polyketide metabolism (Tocci et al., 2018b), and therefore might contain novel antifungal remedies.

Here we report new data on the inhibitory activity of extracts deriving from *H. montanum*, H. *maculatum, H. hirsutum*, and *H. hircinum* subsp. *majus* and additional data on the activity of *H. perforatum* on the growth of clinical isolates of *Candida* spp. The crude extracts obtained from *H. maculatum, H. montanum, H. hirsutum*, and *H. perforatum* showed a moderate antifungal activity against *C. albicans*, in the range of 250–500 μg/ml, good activity against *C. lusitaniae* (64–125 μg/ml) and no or only weak activity against *C. parapsilosis, C. tropicalis and C. glabrata*. Our data are in accordance with those of Radulović et al., 2007, showing that extracts of *H. hirsutum, H. maculatum*, and *H. perforatum* exerted comparable antifungal activity against *C. albicans* using the disk diffusion method assay. Discordant data are reported in Cecchini et al., 2007 which found no activity of extracts obtained from *H. hirsutum* or *H. montanum* against *C. albicans*. Most interesting, the antifungal screening of crude extracts unveiled the promising anti-*Candida* properties of *H. hircinum* subsp. *majus*. The test showed that extracts obtained from this species are able to inhibit 50% of growth of *C. albicans* at concentrations that are fourfold to eightfold lower than other *Hypericum* extracts and with a more pronounced activity against *C. parapsilosis* and *C. lusitaniae*. Moreover *H. hircinum* methanolic extract was the only treatment able to inhibit the growth of *C. tropicalis* and *C. glabrata*. These results are important especially in the light of the increasing incidence of infections caused by emerging *Candida* pathogens like *C. glabrata, C. parapsilosis, C. tropicalis*, and *C. krusei*, which are less susceptible to the commonly used antifungal drugs (Tortorano et al., 2006; Papon et al., 2013). Previous data on the antifungal activity of *H. hircinum* are scarce and limited to susceptibility tests against just a single reference strain of *C. albicans*. Using the disk diffusion test method Pistelli et al., 2000 found no antifungal activity against the tested strain, while Cecchini et al., 2007 reported a higher activity of *H. hircinum* subsp. *majus* in comparison to other *Hypericum* species.

From a chemical point of view, the extracts applied as treatments in our tests have been deeply characterized by investigating the presence of 52 phenolic compounds. In general, the most abundant classes of detected compounds were flavonols, benzoate and cinnamates (chlorogenic acid and neochlorogenic acid), and flavan-3-ols. The major metabolite in all the extracts was the flavonol hyperoside, ranging from 4940 ± 66,3 μg/g in *H. montanum* to 9306 ± 385 μg/g in *H. hircinum* subsp. *majus*, and very abundant were the neochlorogenic acid and the flavan-3-ol epicatechin. Hyperoside is known for its anticancer, anti-inflammatory and antimicrobial properties (Ogawa et al., 2014). The extract of *H. hircinum* subsp. *majus*, showed several chemical peculiarities. It contained the highest amount of hyperoside, higher amounts of chlorogenic acid, quercetin-3-glucuronide, kampferol-3-glucuronide, catechin, epigallogatechin, gallocatechin, and no traces of the phloroglucinol hyperforin and of the naphtodianthrone hypericin.

In the light of its pronounced growth inhibition properties against *Candida* spp. and considering the ethnobotanical knowledge available for this species, *H. hircinum* subsp. *majus* has been further subjected to a deeper antifungal screen. *H. hircinum* is a shrub native to the areas of the Mediterranean Basin comprising Greece and Turkey (Euro+Med, 2006). The infusion of its aerial part is applied in the traditional medicine to treat chronic catarrhal affections, asthma, sore throats and cough (Pieroni et al., 2004). Recent scientific evidences indicate that fungi of the phyla Basidiomycetes and Ascomycetes (e.g., *Bjerkandera, Aspergillus, Candida*, and *Saccharomyces*) are causative agents of these pathologies (Raza et al., 2017).

To investigate whether the beneficial effect of *H. hircinum* treatments may be attributed to its antifungal activity, we studied, for the first time, the activity of extracts obtained from the aerial parts of the plant with a hydroalcoholic solution and, in accordance with the traditional preparations, by infusion in boiling water. Additionally, we characterized the chemical profile. The assay was performed on a broad panel of clinical *Candida* strains, a strain of *Aspergillus glaucus* and a strain of *Penicillium paneum* (**Table 3** and Supplementary Table S3). The extract obtained from the herbal tea inhibited 50% of the growth of all tested fungi, showing strong activity against both fluconazole sensitive and resistant *Candida* strains and moderate activity against *Aspergillus* and *Penicillium* strains. The chemical profile revealed an enrichment in the polyphenol content in comparison with the crude methanolic extract. Particularly abundant (content higher than 40 μg/g extract) were caffeic acid, chlorogenic, neochlorogenic and cryptochlorogenic acid, quercetin, quercetin-3-glucoside, quercetin-3-glucuronide, kaempferol-3-glucuronide, all the flavan-3-ols investigated and extraordinary high levels of hyperoside were detected. Even if the bioactivity may be due to the synergistic action of the phytocomplex, it appears reasonable to exclude caffeic acid, chlorogenic acid, quercetin-3-glucuronide, kaempferol-3-glucuronide, and gallocatechin as secondary metabolites to which the antifungal activity may be attributed, since these compounds are not present in the other *Hypericum* extracts showing growth inhibitory properties on *Candida* strains. Also, cryptochlorogenic acid, neochlorogenic acid quercetin, quercetin-3-glucoside, epicatechin, have been detected in all the crude extract, but they were less present in *H. hircinum* subsp. *majus* and thus their presence cannot be considered correlated to the bioactivity. According to our data a positive correlation between the amount of catechin, epigallocatechin, procyanidin B_1_ and hyperoside and the antifungal activity exerted can be individuated. The anti-*Candida* properties of flavan-3-ols, and in particular of catechin and epigallocatechin, are well known and documented (Hirasawa and Takada, 2004; Saito et al., 2013). Nevertheless, our data suggest that the hyperoside, being detected in such high amounts in the extract obtained by the infusion of *H. hircinum* subsp. *majus* in water, may play a fundamental role in the observed fungal growth inhibition properties. The hypothesis is supported by studies proposing hyperoside as a lead for the development of new fungicides (Li et al., 2005).

Finally, the aqueous extract of *H. hircinum* subsp. *majus* showed another promising characteristic exploitable for the development of a new antifungal therapy. When tested at high concentrations on human PBMCs and human skin fibroblast Hs27cell lines, no cytotoxicity was detected.

## Conclusion

Our work reports a comprehensive phenolic profile of five European *Hypericum* species, highlighting the presence of important exploitable bioactive metabolites. The infusion of *H. hircinum* subsp. *majus* used in the popular medicine for the treatment of respiratory tract affections has been chemically and biologically investigated for the first time. The chemical analysis unveiled a new source of the bioactive metabolite hyperoside that might be involved in the observed bioactivity of the extract. The biological tests suggest that the beneficial effects of the traditional remedies based on *H. hircinum* could be attributed to its antiseptic, and in particular to its antifungal properties against fungal pathogens of the respiratory tract. Moreover, the lack of cytotoxicity indicates the presence of exploitable and promising antimycotic principles for the development of an antifungal therapy with limited cytotoxic side effects. Hence it encourages future studies to assess the effective therapeutic properties of *H. hircinum* subsp. *majus* with particular attention to the treatment of fungal-associated pathologies of the respiratory tract.

## Ethics Statement

PBMCs were obtained from buffy coats prepared from blood donations collected at the Centro Trasfusionale c/o Ospedale Santa Chiara, Trento, after donor’s signed informed consent. Study procedures were approved by the Comitato Etico per le Sperimentazioni Cliniche, Azienda Provinciale per i Servizi Sanitari di Trento on the 12th February 2015.

## Author Contributions

NT, TW, and FM conceived the study and wrote the manuscript with contributions from all authors. NT, DP, FF, and TW performed the experiments. DI identified the species. NT, DP, and TW analyzed the data. NT drafted the manuscript. All authors approved the final manuscript.

## Conflict of Interest Statement

The authors declare that the research was conducted in the absence of any commercial or financial relationships that could be construed as a potential conflict of interest. The reviewer LA declared a shared affiliation, though no other collaboration, with one of the authors DI to the handling Editor.

## Abbreviations

CFU: colony forming units
GM: geometric mean
LC-MS: liquid chromatography-mass spectrometry
MIC: minimal inhibitory concentration
