# Supplementary material for: Extracts From Hypericum hircinum subsp. majus Exert Antifungal Activity Against a Panel of Sensitive and Drug-Resistant Clinical Strains
Source: Front Pharmacol. 2018 Apr 20;9:382. doi: 10.3389/fphar.2018.00382 (PMC5932341; doi:10.3389/fphar.2018.00382)
Supplement: Supplementary file 1 [file Table_1.DOCX]

**Table S1. MRM transitions**

| **Metabolite** | **rt (min)** | **ESI mode** | **Cone Voltage** | **MRM transitions** quantifier ion | **MRM transitions** qualifier ion | **Supplier** |
| --- | --- | --- | --- | --- | --- | --- |
| **Hyperoside** | 1,92 | - | 32 | 463,30 → 300,2 (26) | 463,3 → 244,03 (32) | Sigma-Aldrich |
| **Hypericin** | 7,10 | - | 68 | 503,41 → 405,15 (52) | 503,41 → 430,70 (44) | Sigma-Aldrich |
| **Hyperforin** | 5,60 | - | 36 | 535,68 → 312,80 (28) | 535,68 → 383,34 (32) | Sigma-Aldrich |
