# Supplementary material for: Extracts From Hypericum hircinum subsp. majus Exert Antifungal Activity Against a Panel of Sensitive and Drug-Resistant Clinical Strains
Source: Front Pharmacol. 2018 Apr 20;9:382. doi: 10.3389/fphar.2018.00382 (PMC5932341; doi:10.3389/fphar.2018.00382)
Supplement: Supplementary file 2 [file Table_2.docx]

**Table S2. Antifungal activity of crude *Hypericum* extracts**

|  |  | |  | |  |  | | |  | |  |  |  |  |  |  |  |
| --- | --- | --- | --- | --- | --- | --- | --- | --- | --- | --- | --- | --- | --- | --- | --- | --- | --- |
|  | ***H. hircinum*** | | | ***H. maculatum*** | | | | ***H. montanum*** | | | | ***H. perforatum*** | | ***H. hirsutum*** | | **Fluconazole** | |
|  | **MIC_50_** | **MIC_90_** | | **MIC_50_** | | | **MIC_90_** | **MIC_50_** | | **MIC_90_** | | **MIC_50_** | **MIC_90_** | **MIC_50_** | **MIC_90_** | **MIC_50_** | **MIC_90_** |
| ***C. albicans***  **ATCC MYA-2876** | 125±0,013 | >500 | | 250±0,021 | | | >500 | 500±0,01 | | >500 | | 250±0,01 | >500 | 500±0,02 | >500 | 0,125±0,01 | 1±0,05 |
| ***C. albicans* YN7** | 32±0,017 | 250±0,15 | | 250±0,05 | | | >500 | 500±0,19 | | >500 | | 500±0,18 | >500 | 500±0,08 | >500 | 1±0,03 | 2±0,08 |
| **C. *parapsilosis* YB1** | 32±0,008 | 250±0,78 | | 500 | | | >500 | >500 | | >500 | | 125±0,02 | >500 | 500 | >500 | 64±0,023 | >64 |
| ***C. parapsilosis* YB3** | 32±0,011 | 250±0,15 | | 500 | | | >500 | >500 | | >500 | | 125±0,01 | 500 | 500 | 500 | 32±0,02 | >64 |
| ***C. tropicalis* MFB35-1** | 250±0,01 | >500 | | >500 | | | >500 | >500 | | >500 | | >500 | >500 | >500 | >500 | >64 | >64 |
| ***C. tropicalis* RTT037** | 500±0,046 | >500 | | >500 | | | >500 | >500 | | 500 | | >500 | >500 | >500 | >500 | >64 | >64 |
| ***C. lusitaniae* YHS217** | 16±0,050 | 125±0,05 | | 64±0,11 | | | 500 | 125±0,05 | | >500 | | 125±0,03 | >500 | 64±0,03 | >500 | 0,5±0,005 | 2±0,02 |
| ***C. glabrata* MFB004-1** | 250±0,069 | >500 | | >500 | | | >500 | >500 | | >500 | | >500 | >500 | >500 | >500 | 0,13±0,08 | 1±0,01 |

*Data are means of three independent experiments
