# Supplementary material for: Extracts From Hypericum hircinum subsp. majus Exert Antifungal Activity Against a Panel of Sensitive and Drug-Resistant Clinical Strains
Source: Front Pharmacol. 2018 Apr 20;9:382. doi: 10.3389/fphar.2018.00382 (PMC5932341; doi:10.3389/fphar.2018.00382)
Supplement: Supplementary file 3 [file Table_3.docx]

**Table S3. Antifungal activity of *H. hircinum* subps. *majus***

| **Strain** | **MeOH** | | **80%EtOH** | | **Infusion** | | **Fluconazole** | |
| --- | --- | --- | --- | --- | --- | --- | --- | --- |
|  | **MIC_50_** | **MIC_90_** | **MIC_50_** | **MIC_90_** | **MIC_50_** | **MIC_90_** | **MIC_50_** | **MIC_90_** |
| ***C. albicans* ATCC MYA-2876** | 125±0,013 | >500 | 125±0,01 | >500 | 125±0,02 | >500 | 0,125±0,01 | 1±0,05 |
| ***C. albicans* YN7** | 32±0,017 | 125±0,14 | 64±0,19 | >500 | 64±0,46 | 250±0,02 | 1±0,03 | 2±0,08 |
| ***C. albicans* YHS254** | 16±0,56 | 125±0,78 | 125±0,01 | 500±0,59 | 125±0,02 | 250±0,12 | 32±0,01 | >64 |
| ***C. albicans* YHS89** | 16±0,51 | 64±0,02 | 32±0,44 | 250±0,32 | 16±0,08 | 125±0,05 | 64±0,02 | >64 |
| ***C. glabrata* MFB004-1** | 16±0,85 | 64±0,54 | 64±0,65 | 250±0,54 | 64±0,24 | 250±0,21 | 0,13±0,01 | 1±0,01 |
| ***C. lusitaniae* YHS217** | 16±0,05 | 125±0,78 | 32±0,02 | 250±0,78 | 64±0,36 | 250±0,16 | 0,5±0,01 | 2±0,02 |
| ***C. lusitaniae* YHS72** | 16±0,18 | 64±0,94 | 125±0,32 | 250±0,45 | 16±0,32 | 125±0,84 | 0,25±0,01 | 1±0,05 |
| ***C. tropicalis* MFB035-1** | 250±0,01 | >500 | 500±0,75 | >500 | 250±0,44 | >500 | >64 | >64 |
| ***C. tropicalis* RTT 037-1** | 500±0,05 | >500 | 250±0,34 | >500 | 125±0,15 | >500 | >64 | >64 |
| ***C. parapsilosis* YHS133** | 16±0,18 | 250±0,15 | 125±0,08 | >500 | 16±0,87 | 64±0,14 | >64 | >64 |
| ***C. parapsilosis* YHS312** | 16±0,14 | 125±0,46 | 125±0,04 | 500±0,85 | 125±0,03 | 500±0,95 | >64 | >64 |
| ***C. parapsilosis* YHS301** | >500 | >500 | >500 | >500 | 250±0,17 | >500 | >64 | >64 |
| **C. *parapsilosis* YB1** | 32±0,05 | 250±0,27 | 250±0,25 | 500±0,78 | 125±0,08 | 500±0,15 | 64±0,02 | >64 |
| ***C. parapsilosis* YB3** | 32±0,01 | 250±0,85 | 250±0,17 | 500±0,93 | 125±0,09 | 500±0,89 | 32±0,02 | >64 |
| ***A. glaucus*  YHS165** | >500 | >500 | >500 | >500 | 250±0,19 | >500 | 0,25 ±0,05 | 16±0,08 |
| ***P. paneum* YHS245** | >500 | >500 | >500 | >500 | 64±0,02 | 500±0,64 | 0,125±0,06 | 4±0,07 |

*Data are means of three independent experiments
